# Household Macronutrient Prices and Livestock Health in Western Kenya

**DOI:** 10.3389/fvets.2020.547348

**Published:** 2020-11-13

**Authors:** Alexander J. Kappes, Thomas L. Marsh

**Affiliations:** Washington State University, Pullman, WA, United States

**Keywords:** nutrient consumption, production, development, livestock health, policy, nutrient costs

## Abstract

Understanding food insecurity issues is in part contingent on understanding food consumption and its costs. We develop estimates of protein, lipid, and carbohydrate macronutrient consumption from household food consumption in western Kenya. We then calculate the shadow price per gram of macronutrient consumption as a share-weighted expense-consumption ratio. Using household bovine, goat, and sheep livestock health observations linked to each household, we analyze the association between livestock illness and macronutrient prices. We find that on average carbohydrates have a 75% budget share, with protein at 14% and lipids at 11%. Average macronutrient shadow prices are 0.0936 Ksh/g for carbohydrates, 0.4373 Ksh/g for protein, and 0.5938 Ksh/g for lipids. Average village-level livestock illness occurrences have significant effects on macronutrient shadow prices. Increasing average bovine illness at the village level by one additional case results in a marginal increase of the shadow prices of protein, lipids, and carbohydrates by 0.11, 0.12, and 0.03 (Ksh/g), respectively. Associated marginal impacts of sheep illness occurrence on protein, lipid, and carbohydrate shadow prices (Ksh/g) are 0.1405, 0.182, and 0.0455, respectively. This exploratory analysis provides empirical evidence that livestock illness is associated with increased macronutrient shadow prices, and hence the costs of available energy consumption. These results help guide policy instruments focused on market forces of nutrient consumption and its relationship with livestock health in undernourished areas with smallholder farming systems.

## Introduction

Sub-Saharan Africa has the lowest per person per day energy availability ranking worldwide (1). Availability, access, and utilization commonly define the pillars of food security (2). Food insecure households experience a failure of these pillars and consume an insufficient amount of nutrients. Negative health effects associated with malnourishment result in a failure to meet both cognitive and physical growth potential, which is linked to reductions in educational attainment and future earnings (3). The socio-economic impacts of food insecurity and its associated health effects consequently create an indirect effect of intergenerational poverty transmission (4). Increasing energy availability helps to provide opportunity for sufficient nutrient consumption, ultimately promoting health and welfare in underdeveloped areas.

Livestock represent a critical component in smallholder farming systems in underdeveloped areas (5). The agricultural household characterizes this smallholder farming system with production allocated toward subsistence needs and local market supply. The consumption of crops and animal sourced foods depends on the productivity of livestock within these systems. Livestock health directly affects productivity in terms of draft capability and meat production, which in turn affects nutrients available for consumption. Energy availability, and thus nutrient consumption, is partially dependent on the efficiency of livestock meat and crop production. Agricultural households with healthy livestock use feedstuff more efficiently for meat production and also use less human labor to augment unhealthy livestock crop production. Healthy livestock promote increased energy availability for the agricultural household and for local markets they supply, and also allow household members to allocate time toward economic activities other than crop production.

Nutrient consumption flows and the cost of nutrients provide important information on availability, access, and utilization of nutrients. The objective of this paper is to provide an exploratory analysis of rural western Kenya household macronutrient consumption and the association between livestock health and macronutrient prices, and consequently the association with costs of macronutrient consumption. Using household food consumption and livestock health observations, we accomplish this object by firstly converting food consumption into macronutrient consumption, and then secondly calculate per-gram macronutrient shadow prices for providing a measurement of macronutrient consumption costs, and lastly we specify an additive model relating livestock health to macronutrient consumption costs for evaluating their statistical association. The associated marginal effects of variation in livestock health on the variation of macronutrient consumption costs is evaluated through the additive model in a descriptive environment. Our findings contribute to current human health and development literature by providing quantitative measurements of macronutrient consumption costs and price impacts associated with variation in livestock health. This information benefits the construction of food consumption interventions particularly and nutrition policy in general in malnourished areas with representative smallholder farming systems.

## Data and Methods

The ongoing population-based animal syndromic surveillance (PBASS) socioeconomic system provides the data used for this study. This system enables health and economic analysis (6) through the collection of individual-level data from villages located in the rural western Kenya region. Data informing this study spans from February 2013 to June 2016 and consists of observations on household-level characteristics, food consumption, and expense, as well as livestock symptom presentation. Data collection occurs on a monthly basis through interviews gathering representative information on average food consumption and expense based on a 7-day recall, and observed livestock symptoms since last visit informed by both the household and veterinary professionals who visit the households. See (6) for research protocols and data collection details. We focus on bovine, sheep, and goats. While we recognize that these livestock species are not the only livestock used by households in this region, they are the livestock for which symptomatic health observations are available and linked directly to each household and its consumption patterns. We observe 588 unique livestock-owning households with ~14 observations each over the sample period.

The United States Department of Agriculture (USDA) Food Composition Databases are used to convert food consumption into macronutrient consumption (1). Using macronutrient composition data for all food items consumed in our sample, we estimate household macronutrient intake as

$$\begin{array}{l} N_{h}=\overset{L}{\underset{l}{\sum }}\overset{J}{\underset{j}{\sum }}\gamma_{l}F_{j} \end{array}$$

where *N* represents total macronutrient consumption in grams for household *h*, γ represents the conversion factor for macronutrient *l* as the collection of protein, lipid, and carbohydrate, and *F* represents food consumption for food item *j*. The estimator for consumption in grams of individual macronutrients *N*_*l, h*_ is characterized by removing the summation over *L* in the calculation of total macronutrient consumption above, providing $N_{l,h}=\;\overset{J}{\underset{j}{\sum }}\gamma_{l}F_{j}$.

The conversion factor γ for each macronutrient is provided by the USDA Food Composition Database in terms of macronutrient quantities per serving size of food item *j*. Food consumption *F* is converted to a measurement of total servings consumed, where the conversion factor is used to compute total macronutrient consumption for that particular food item.

The associated costs of macronutrient consumption are evaluated through the estimation of shadow prices, which reveal market information for goods whose prices are not directly observable. Each shadow price is estimated as a share-weighted expense-consumption ratio

$$\begin{array}{l} P_{l,h}=\theta_{l,h}\left(\frac{E_{h}}{N_{l,h}}\right) \end{array}$$

where *P* represents the per-gram shadow price household *h* pays to consume macronutrient *l*, θ represents the household's consumption share for each macronutrient, and *E* represents household food expense, which is defined as the cost a household pays for purchasing food items in a market environment. It is important to note that due to the nature of farming systems in this area, a proportion of total food items consumed observed in the data can come from home production of that item. An example is production of cow milk by a household cow with milk consumed by the household, where a proportion of total cow milk consumed is accounted for by their cow's milk production. However, this information is accounted for when estimating shadow prices of the macronutrients derived from the household's home-produced food item. To extend the previous example the shadow prices of macronutrients derived from home-produced cow milk reflect information on the cost of macronutrient consumption for that household producing some of its own food items. Compared to a household that does not produce cow milk, the household that does produce it realizes lower shadow prices for macronutrients derived from its consumption as its expense for that consumption is less. The cow milk example extends directly to all food items produced at home. We assume that a household is rational in their decision to produce a food item at home for a proportion of their total consumption of that food item, where the associated costs of producing that item are lower than purchasing that item in a market environment. The nature of food items is only important insofar as estimating macronutrient consumption. Discrimination between food items consumed takes place through varying consumption levels of macronutrients that make up these food items.

The statistical relationship between household macronutrient consumption cost and livestock health is explored in an additive regression framework. Additive, or linear model specification in general, evaluates systematic variation between a response variable and one or more independent variables related to the response variable (7). Evaluating how variables vary together allows for analysis of a statistical relationship between them. Livestock illness directly affects livestock production, which directly affects energy availability through supply in a subsistence and local market environment. We avoid placing constraints on the analysis by not specifying probability distributions or prior beliefs in the data generating process as called for in Bayesian environments (8), nor do we explore more elaborate functional forms due to risk of misspecification (9). Generally speaking, a linear model can be interpreted as a first order approximation to any functional form. Our analysis is an initial exploratory evaluation of estimating the statistical association between livestock health and the cost of macronutrient consumption.

Market forces influence household consumption patterns, which are accounted for by realized budget shares of consumption. Allocation of constrained resources to food items take into account the opportunity cost of consumption for competing goods. Consumption pattern decisions become a function of cost, income, and evaluation of welfare derived from consumption of competing goods. The effect of competing goods is known by the household when making consumption decisions, but is unobserved in available data (10). The stochastic error term in statistical models evaluating consumption decisions accounts for this unobserved effect.

We extend measurement of consumption patterns by estimating macronutrient shadow prices as a deterministic function of food consumption budget shares, which takes into account expense and implicitly places a lower bound on household income. Relating systematic variation in livestock illness to costs of macronutrient consumption takes place through livestock production. Existing literature explores the importance of livestock health in production by evaluating optimal control programs for decreasing mortality using dynamic programming methods (11), and through cost-benefit analysis showing high return on investment for animal health programs increasing production efficiency by decreasing the impacts of disease (12). Aside from the significance of livestock health in production, existing literature also explores the importance of livestock health in human welfare. There exists positive effects of increased livestock health on access to animal sourced foods and income generation for greater purchasing power in livestock-owning households (6). While livestock production is in part constrained by disease (13), and thus health, its contribution to income is significant in rural Sub-Saharan African households, which allows further access to animal sourced foods through purchasing power (14). Evaluating the systematic variation between macronutrient shadow prices, in which consumption patterns are subsumed, and livestock health through statistical association is supported by previous research evaluating links between livestock health, production, and human welfare through consumption and income.

While production methods are important in evaluating production outcomes, this study is interested in livestock health outcomes and its relationship with macronutrient shadow prices of household food consumption. To this end, livestock production methods and constraints are subsumed in livestock health outcomes. An example of this includes availability of feed and water. Inadequate availability or access to these production factors leads to malnourishment, poor production, and illness, with the latter of these outcomes observed through data.

Livestock health observations are used to characterize average livestock illness occurrence at village levels. The representative aggregate measure of livestock illness occurrence is used as an instrumental variable for determining household-level livestock illness occurrence due to a potentially endogenous effect between the household's food consumption expense and their production of animal sourced foods and crops. The presence of endogeneity is evidenced in literature examining household livestock production for subsistence and income generation in underdeveloped areas (6, 14). As food consumption expense is determined in part by household agricultural production, which is in part determined by livestock production and is influenced by livestock health, there exists simultaneity between food consumption expense and livestock illness at the household level. The simultaneous relationship leads to endogeneity problems in parameter estimation, resulting in estimation bias from error term correlation with included regressors (9). We remove simultaneity at the household level by smoothing livestock illness across all households and aggregate it to a representative figure at the village level, where any one household does not influence measurements at this level. This is accomplished by using average livestock illness occurrence for each village. This transformation still relates livestock health to cost of macronutrient consumption at the household level because of the homogeneity of household livestock production and livestock illness occurrence within villages, and lack of market mobility across villages.

Ordinary least squares error term assumptions of having an expected value of zero and being uncorrelated with included regressors are established through the use of average village livestock illness as an instrumental variable, which permits the evaluation of a statistical association between cost of macronutrient consumption and livestock illness. Because the number of household members explains consumption expense and must be controlled for due to its availability (7), it is also included in the additive model. The model relating livestock illness to cost of macronutrient consumption is specified as

$$\begin{array}{l} P_{l,h}=\alpha_{l}+\beta_{1}I_{v,m}+\beta_{2}THM_{h}+\;\varepsilon_{h} \end{array}$$

Let *I* represent average livestock illness occurrence for village *v* in month *m*, *THM* represent total household members for household *h*, and ε represent a random component explaining variation in the macronutrient's shadow price not explained by the included variables *I* and *THM*. The estimable parameters are α, β_1_, and β_2_ with the parameter of interest for livestock health effects being β_1_. Given the panel data structure of sample data, inference on estimated parameters is conducted using a Heteroskedastic and Autocorrelation Consistent covariance matrix (15).

## Results and Discussion

**Figure 1:**
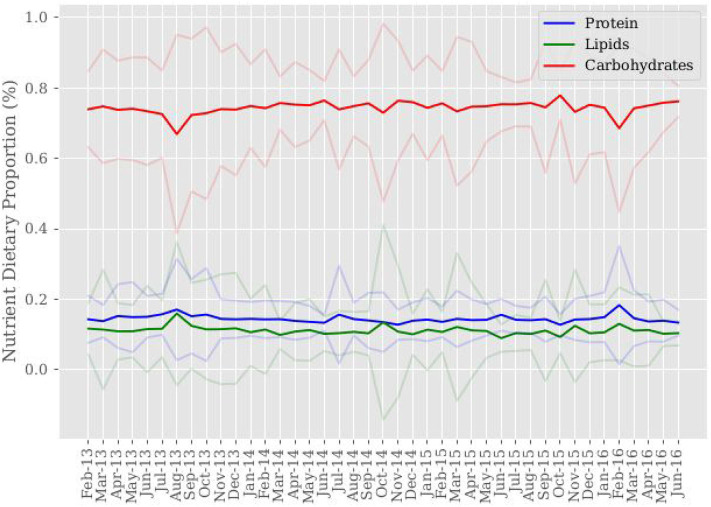
Sample mean of nutrient dietary proportions. Nutrient dietary proportions are computed from nutrient-food item consumption conversions. Mean consumption proportion values are then computed for each month across sample time. 95% confidence intervals around mean consumption proportions are shown in color corresponding to each macronutrient.

Carbohydrates account for the largest proportion of consumed macronutrients with a share of ~0.75 on average, as displayed in Table 1. Figure 1 displays macronutrient consumption share trends over sample time. Noticeable in Figure 1 is a subtle substitution effect between consumption of carbohydrates and consumption of protein and lipids. The substitution effects are more pronounced during the time frames July 2013–September 2013 and January 2016 - March 2016. On average, protein's consumption share is slightly higher at ~0.14 than lipids' consumption share at ~0.11. Our estimates of carbohydrate and protein proportions consumed agree with the range of macronutrient proportions found among adults across three ethnic groups in rural Kenya during a recent study (16). However, our estimate of the proportion of lipids consumed falls below their range of 14.5–30.2% mean estimates across ethnic groups. Differences in diets across ethnic groups contributes to the wide range of macronutrient proportions consumed, with access and availability of nutrients being an important factor.

**Table 1:** Variable summary statistics (*N* = 1078).

	**Dietary proportion**			**Shadow price (Ksh/g)**				
	**Protein**	**Lipids**	**Carbohydrates**	**Protein**	**Lipids**	**Carbohydrates**	**Household livestock illness average**	**Total household members**
Mean	0.1433	0.1116	0.7451	0.4374	0.5938	0.0936	1.1531	4.8692
Std	0.0338	0.0577	0.0736	0.2878	0.4020	0.1525	0.2412	2.3239
Min	0.0066	0.0293	0.0345	0.0095	0.0041	0.0012	0.0000	1.0000
Max	0.5321	0.9588	0.9046	2.8381	4.9541	3.2035	3.0000	17.0000

*Nutrient shadow prices are computed as share-weighted consumption-expense ratios and provide nutrient consumption costs in terms of Ksh/g. Sample data spans February 2013–July 2016. Nutrient Shadow prices are deflated using the February 2013 Kenya CPI. Livestock illness village averages are computed for each time period and averaged over the sampling period, representing the average number of ill livestock per household across all villages*.

Access to and consumption of carbohydrate rich foods is greater than that of consuming food items with a greater nutrient makeup of protein and lipids. The resulting macronutrient consumption shares for carbohydrates and protein are inline with the World Health Organization (WHO) and the Food and Agricultural Organization's (FAO) recommended macronutrient intake proportions for a balanced diet. The recommended range of total carbohydrate consumption is 0.55–0.75, with a range for protein of 0.10–0.15, and a range for total fats of 0.15–0.3. From the resulting macronutrient proportions it is estimated that on average, an upper bound of 25.5% of macronutrients consumed are from animal sourced foods. Using ranged macronutrient proportions to describe a balanced diet, this area of study sees proportions of carbohydrate and protein consumption at and toward the recommended upper range on average, respectively, with proportions of lipid consumption below the recommended lower range on average. WHO and FAO recommended dietary proportions establish population nutrition goals for the prevention of chronic diseases related to dietary intake (17). Further evidence supports the significance of diet diversification in nutrient rich items for promoting health development outcomes (18). However, it is important to note that while macronutrient intake proportions may fall inline with recommended proportions, with the exception of lipids, the total quantities of nutrients consumed are inadequate in promoting nourishment, as evidenced by the region's lack of energy availability (1).

**Figure 2:**
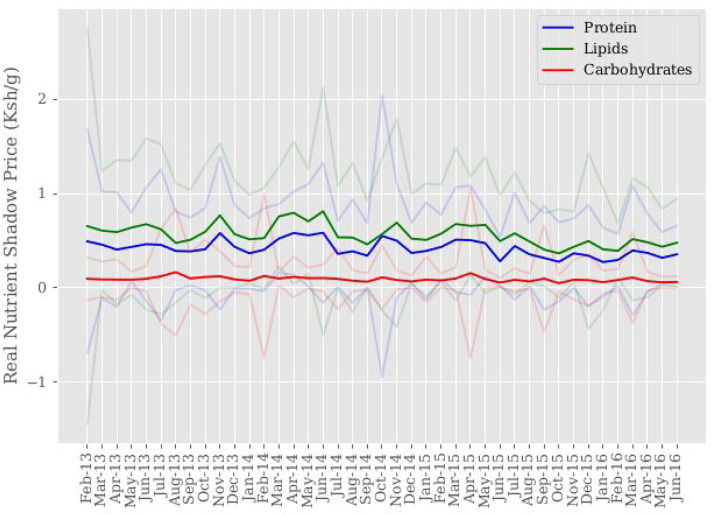
Sample mean of nutrient shadow price (Ksh/g). Mean shadow prices for each nutrient are computed for each month across sample time. 95% confidence intervals around mean macronutrient shadow prices are shown in color corresponding to each macronutrient shadow price.

On average, real macronutrient shadow prices (Ksh/g) are estimated to be 0.094 for carbohydrates, 0.437 for protein, and 0.594 for lipids, as displayed in Table 1. Figure 2 displays mean macronutrient shadow price variation over sample time. Carbohydrate shadow prices are more stable across sample time than the shadow prices for protein and lipids. The standard deviation for mean carbohydrate shadow prices is ~0.15, which is compared to the standard deviations of mean protein and lipids shadow prices at ~0.29 and 0.4, respectively. Evidence of shadow price stability across time is seen in Figure 2 with carbohydrate shadow prices revealing a relatively flat trend, while shadow prices for protein and lipids experience larger fluctuations.

Stability of availability and access, made possible through lower household cost and cost variation, results in consumption dense in carbohydrate rich foods. Lower consumption shares of protein and lipids are reflected through increased household costs. Consumption of lipids is estimated to be the most costly, while also having the most instability due to its larger variation. Framing this information in terms of market effects leads to speculation that lower lipid consumption is associated with increased cost of consumption as a result of lipid availability.

Utilization of protein and lipids rich food items is contingent on their availability and a household having access to them, in terms of being able to purchase or produce the food items. Animal sourced foods predominantly supply protein and lipids rich food items, which aid in cognitive and physical development (19). Understanding the determinants of availability and access to animal sourced foods then becomes a key issue in ensuring opportunities for both having balanced diets and a sufficient amount of nutrients for the energy needed to promote human health. Livestock health is one of the determinants of macronutrient availability through agricultural production, and information on a household's access to these nutrients is evaluated through the marginal association between livestock health and consumption costs.

Per-month village-level livestock illness occurrence results in each household experiencing an approximate average of 1.15 illness occurrences in their own livestock each month. Livestock illness occurrences are defined as observed symptoms of reproductive, respiratory, digestive, urogenital, muscle, skin, and/or nerve disorders. The largest occurrence of observed bovine disorders are accounted for by digestive and skin disorders at rates of 0.67 and 0.32, respectively. Nerve and muscle disorders closely follow at rates of 0.27 and 0.24, respectively. Reproductive disorders are least observed at a rate of 0.005. The largest occurrence of observed goat disorders are accounted for by digestive and reproductive disorders at rates of 0.57 and 0.55, respectively. Respiratory disorder occurrence follows at a rate of 0.37. Nerve disorder is the least observed at a rate of 0.014. The largest occurrence of observed sheep disorders are accounted for by digestive and muscle disorders at rates of 0.77 and 0.29, respectively. Skin and respiratory disorders closely follow at rates of 0.14 and 0.13, respectively. Reproductive disorders are the least observed at a rate of 0.003. As a reference point, rates of general illness for bovine, goat and sheep are 0.55, 0.47, and 0.36, respectively. On average, households are made up of approximately five members.

**Figure 3:**
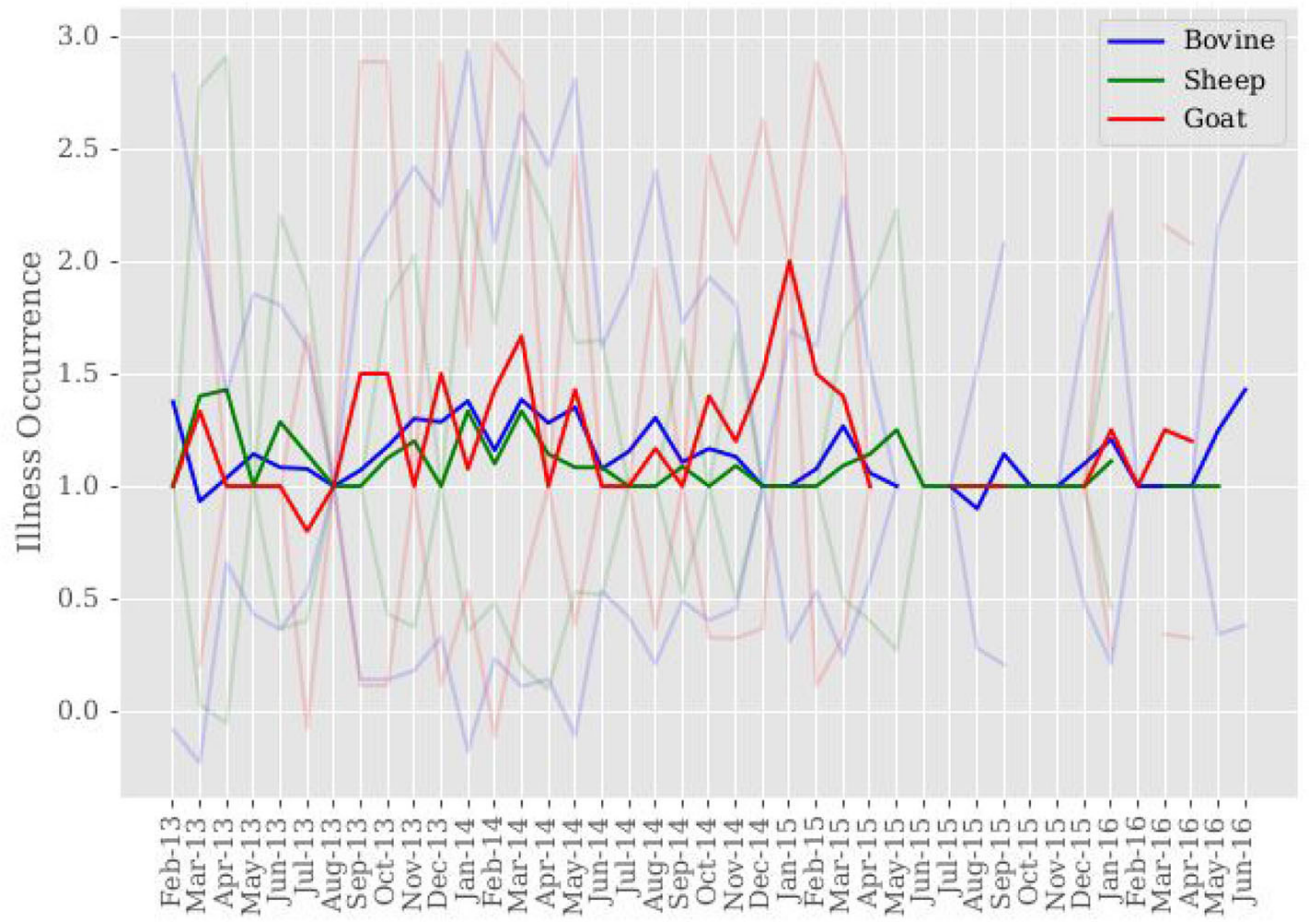
Sample mean of livestock illness occurrence. Mean livestock illness occurrence is computed for each month across sample time. 95% confidence intervals around mean livestock illness occurrence are shown in color corresponding to each livestock species. Breaks in data correspond to having no household livestock observations. It is important to note this does not result in having no livestock illness occurrence, only that it is unobserved.

Figure 3 presents average livestock illness occurrence across sample time aggregated to the sample level, not village level. Breaks in data correspond to having no household observations for that period when matching households across sample time on variables of interest when constructing the data set used for this study. We cannot say there is no livestock illness during these periods, only that it is unobserved. With the exception of February-April 2013, Bovine and sheep illness occurrences appear to be positively correlated across sample time. Visual inspection of Figures 1–3 shows comparisons in trends of macronutrient proportions, shadow prices, and livestock illness occurrences. Notable periods include July-September 2013 and November-December 2014 where trends show slight increases in protein and lipid consumption with decreases in their shadow prices and livestock illness occurrence for bovine and sheep. It is speculated that the link between these trends is livestock health impact on food availability through production, which then impacts consumption decisions through cost.

**Figure 4:**
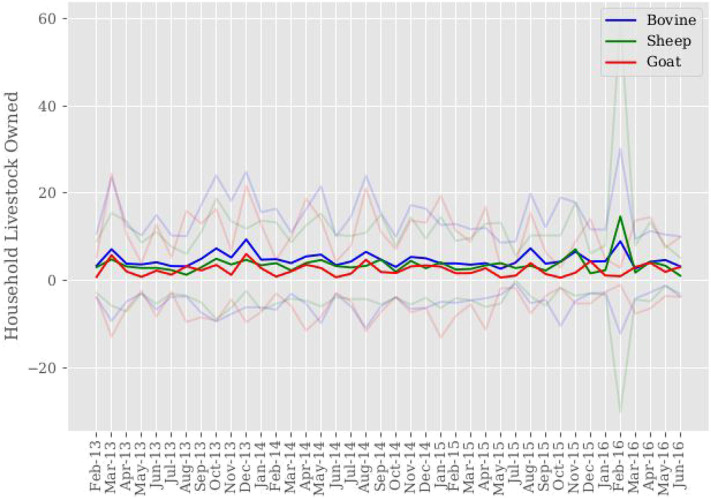
Sample mean of household livestock ownership. Mean household livestock ownership is computed for each month across sample time. 95% confidence intervals around mean livestock ownership are shown in color corresponding to each livestock species.

Figure 4 presents average livestock ownership by species over sample time. Aggregated across households and sample time, households own on average 4.77 head of cattle, 3.49 sheep, and 2.51 goats. Livestock ownership appears stable across sample time, notwithstanding the increase in cattle and sheep ownership during the February 2016 time frame. It is speculated that household culling decisions give way to replacement decisions.

### Regression Outcomes

Table 1 provides summary statistics of all model variables. Tables 2–4 provide the associated marginal effects between bovine, sheep, and goat health and macronutrient shadow prices.

**Table 2:** Bovine health effects on nutrient shadow prices.

**Dependent**	**Independent**	**Coefficient**	**Std errors**	***t-*values**	***Pr*(>|*t*|)**	
Protein	Intercept	0.3197	0.0617	5.1796	0.0000	***
	Livestock illness avg	0.1113	0.0507	2.1934	0.0283	**
	Total HH members	−0.0044	0.0058	−0.7659	0.4438	
Lipids	Intercept	0.4172	0.0934	4.4678	0.0000	***
	Livestock illness avg	0.1210	0.0726	1.6658	0.0958	*
	Total HH members	0.0047	0.0071	0.6541	0.5131	
Carbohydrates	Intercept	0.0647	0.0162	3.9961	0.0001	***
	Livestock illness avg	0.0341	0.0142	2.4110	0.0159	**
	Total HH members	−0.0031	0.0024	−1.2785	0.2011	

OLS specified regression results for bovine livestock health effects on costs of nutrient consumption at the household level. Covariance matrix is estimated using the (20) Heteroskedastic and Autocorrelation Consistent Covariance Estimator. {***, **, *} Significant at the {0.01, 0.05, 0.1} level. The adjusted R squared for protein, lipids, and carbohydrates models are 0.005, 0.002, and 0.005, respectively.

**Table 3:** Sheep health effects on nutrient shadow prices.

**Dependent**	**Independent**	**Coefficient**	**Std errors**	***t-*values**	***Pr*(>|*t*|)**	
Protein	Intercept	0.3072	0.0627	4.8987	0.0000	***
	Livestock illness avg	0.1405	0.0601	2.3364	0.0195	**
	Total HH members	−0.0047	0.0064	−0.7417	0.4582	
Lipids	Intercept	0.3334	0.0871	3.8271	0.0001	***
	Livestock illness avg	0.1820	0.0675	2.6980	0.0070	***
	Total HH members	0.0133	0.0081	1.6417	0.1006	
Carbohydrates	Intercept	0.0706	0.0182	3.8862	0.0001	***
	Livestock illness avg	0.0455	0.0263	1.7281	0.0840	*
	Total HH members	−0.0055	0.0045	−1.2164	0.2238	

OLS specified regression results for bovine livestock health effects on costs of nutrient consumption at the household level. Covariance matrix is estimated using the (20) Heteroskedastic and Autocorrelation Consistent Covariance Estimator. {***, **, *} Significant at the {0.01, 0.05, 0.1} level. The adjusted R squared for protein, lipids, and carbohydrates models are 0.005, 0.002, and 0.005, respectively.

**Table 4:** Goat health effects on nutrient shadow prices.

**Dependent**	**Independent**	**Coefficient**	**Std Errors**	***t-*values**	***Pr*(>|*t*|)**	
Protein	Intercept	0.5620	0.1400	4.0134	0.0001	***
	Livestock illness avg	−0.0363	0.0605	−0.5994	0.5489	
	Total HH members	−0.0088	0.0152	−0.5810	0.5613	
Lipids	Intercept	0.7038	0.2005	3.5097	0.0004	***
	Livestock illness avg	−0.0495	0.1005	−0.4925	0.6223	
	Total HH members	−0.0009	0.0195	−0.0459	0.9634	
Carbohydrates	Intercept	0.1469	0.0506	2.9045	0.0037	***
	Livestock illness avg	−0.0373	0.0270	−1.3785	0.1681	
	Total HH members	0.0013	0.0074	0.1749	0.8612	

OLS specified regression results for bovine livestock health effects on costs of nutrient consumption at the household level. Covariance matrix is estimated using the (20) Heteroskedastic and Autocorrelation Consistent Covariance Estimator. {***, **, *} Significant at the {0.01, 0.05, 0.1} level. The adjusted R squared for protein, lipids, and carbohydrates models are 0.005, 0.002, and 0.005, respectively.

Average bovine illness occurrence is associated with significant marginal increases in shadow prices for carbohydrates, protein, and lipids at the 0.1 level. Unit increases in average bovine illness occurrence is associated with similar increases of ~0.11 and ~0.12 Ksh/g in the shadow prices of protein and lipids, respectively. The associated increase in the shadow price for carbohydrates is ~0.03 Ksh/g when average bovine illness occurrence increases by unit amounts. Average sheep illness occurrence also shows significant associated marginal increases in shadow prices for all macronutrients. The shadow prices for protein and lipids again experience the largest associated increases by ~0.14 and ~0.18 Ksh/g when average sheep illness occurrence increases by unit amounts, with the shadow price for carbohydrates having an associated increase of ~0.05 Ksh/g. Average goat illness occurrence does not show any significant associated marginal changes in macronutrient shadow prices.

Empirical elasticity distributions are calculated for bovine and sheep illness occurrences and reveal associated responses in macronutrient shadow prices to livestock illness events. A 50% empirical interval is used to convey information on the central mass of elasticity values falling between the 25th and 75th percentiles. A percentage change in bovine illness occurrence is associated with a change between 0.23% and 0.53% for the shadow price of protein, 0.19% and 0.44% for the shadow price of lipids, and 0.37% and 0.87% for the shadow price carbohydrates. A percentage change in sheep illness occurrence is associated with a change between 0.27% and 0.56% for the shadow price of protein, 0.26% and 0.54% for the shadow price of lipids, and 0.48% and 1.01% for the shadow price of carbohydrates.

The significant marginal increases associated with bovine and sheep illness occurrence, and macronutrient shadow prices reveal a positive relationship between macronutrient consumption cost and these species' illness occurrences. The adjusted *R*-squared values are reported in the notes section of each table. However, we are not interested in explaining variation in macronutrient shadow prices with the specified model, but rather are interested in evaluating an initial relationship between macronutrient shadow prices and livestock health through statistical association.

The significant association between bovine and sheep health on shadow prices is most notable in the protein and lipids macronutrients that animal sourced foods are rich in. While significant, the associated cost impact of carbohydrate consumption from bovine and sheep livestock health is approximately only a quarter of the associated increase experienced by protein and lipid consumption costs.

Evaluation of associated effects between livestock health and cost of macronutrient consumption provides insight on potential benefits derived from livestock health policy construction. Using the average of five household members, the associated shadow prices for protein, lipids, and carbohydrates during an average of one bovine illness occurrence for households are 0.4088, 0.5616, and 0.0833 Ksh/g, respectively. With no bovine illness occurrences for households, the associated shadow prices for protein, lipids, and carbohydrates are 0.2975, 0.4406, and 0.0492 Ksh/g, respectively. The associated percentage increases in protein, lipid, and carbohydrate shadow prices during bovine illness events are 37.4%, 27.5%, and 69.3%, respectively. Using the same measurement of an average of one sheep illness occurrence for households, associated shadow prices for macronutrients in the same order are 0.424, 0.5819, and 0.888 Ksh/g. With no sheep illness occurrences for households, associated macronutrient shadow prices are 0.2835, 0.3999, and 0.433 Ksh/g. The associated percentage increases in macronutrient shadow prices during sheep illness events are 49.6%, 45.5%, and 105.1% for protein, lipids, and carbohydrates, respectively.

Across all households and all villages average macronutrient consumption in grams for protein, lipids, and carbohydrates over the 7-day recall period is 1,810, 1,562, and 9,879, respectively. Costs savings of protein, lipids, and carbohydrate consumption associated with having an average of no bovine illness events are 201.42, 188.97, and 337.27 Ksh, respectively. Cost savings for macronutrient consumption in the same order associated with having an average of no sheep illness events are 254.3, 284.24, and 449.1 Ksh, respectively. Total cost savings associated with having no bovine illness events becomes 727.66 Ksh, with total cost savings associated with no sheep illness events at 987.64 Ksh. Associated cost savings are across weekly timeframes. As a reference point, for the year 2019 World Data reported an average monthly income in Kenya of 15,842.46 Ksh. Associated cost savings at the monthly level are ~2,910.64 Ksh for households having no bovine illnesses on average, and ~3,950.56 Ksh for households having no sheep illnesses on average.

We consider the most vulnerable households in our sample to be those with no supplemental off-farm income, whose total food expense is greater than the sample's 75th percentile, and whose total household members exceed the average of 5. The associated impact of livestock illness occurrences on macronutrient consumption cost for vulnerable households is greater than the associated impact on the sample's representative household. During bovine illness occurrences associated percentage increases in protein, lipid, and carbohydrate shadow prices are 44.6%, 31.5%, and 89.4%, respectively. Associated percentage increases in macronutrient shadow prices during sheep illness occurrences are 56.9%, 47.9%, and 150.1%, in the same respective order. Compared to a representative household, vulnerable households realize an associated percentage point increase of 7.2, 4, and 20.1 for macronutrient shadow prices during bovine illness occurrences, and 7.3, 2.4, and 45 during sheep illness occurrences.

Costs of policy implementation for increasing livestock health for purposes of increasing human welfare can be compared to the associated cost savings benefits for viability. It is important to note that these measurements only provide initial information on macronutrient cost savings associated with livestock health and should not be used in a definitive, predictive cost savings sense.

## Conclusion

We have estimated macronutrient consumption and their shadow prices, which convey information on the costs households face in consumption decisions. We extend these cost estimates by evaluating variation between livestock health and costs of consumption through the systematic relationship between livestock health and production, and costs of macronutrient consumption and production. We have empirically shown a significant association between bovine and sheep health and the cost of macronutrient consumption. Proper nutrient availability, access, and utilization ensure nutritional requirements for healthy development are met. Increasing the costs of macronutrient consumption in already resource-stricken environments negatively impacts access to these nutrients, whereby decreasing the costs of macronutrient consumption facilitates increased consumption. The positive link between increased livestock health and production aids increases in nutrient availability, helping to decrease costs of macronutrient consumption and make access easier for households.

While our consumption proportion estimates agree with FAO and WHO balanced-diet ranges for carbohydrates and protein, and fall below the recommended range for total fats, current total nutrient consumption in our area of study is inadequate in providing levels of energy consistent with nourished environments. The promotion of health and future well-being is influenced by availability, access, and utilization of these macronutrients. Addressing initiatives seeking to understand macronutrient consumption and mechanisms that increase consumption can in part focus on areas associated with livestock health outcomes for developing areas representative of smallholder farming systems.

## Data Availability Statement

The datasets presented in this article are not readily available because data collection is ongoing as part of the Population Based Animal Syndromic Surveillance System and is property of agencies conducting the survey. Requests to access the datasets should be directed to alexander.kappes@wsu.edu.

## Author Contributions

AK and TM contributed equally in model design, testing, and writing. All authors contributed to the article and approved the submitted version.

## Conflict of Interest

The authors declare that the research was conducted in the absence of any commercial or financial relationships that could be construed as a potential conflict of interest.
